# Pulmonary Metastases from a Hemangiopericytoma/Solitary Fibrous Tumor Spectrum Neoplasm in a Patient with a Poorly Documented Thigh Tumor: A Case Report

**DOI:** 10.3390/reports9030274

**Published:** 2026-08-16

**Authors:** Justina Antonela Dragomir, Alexandru Stoichiță, Silviu Gabriel Vlăsceanu, Radu Matache, Beatrice Mahler

**Affiliations:** 1 Clinical Department 2, Marius Nasta Institute of Pneumophtiziology, 050159 Bucharest, Romania; justina.dragomir@umfcd.ro (J.A.D.); radu.matache@gmail.com (R.M.); beatrice.mahler@umfcd.ro (B.M.); 2 Department of Pulmonology, Carol Davila University of Medicine and Pharmacy, 050474 Bucharest, Romania; 3 Clinical Research Department, Marius Nasta Institute of Pneumophtiziology, 050159 Bucharest, Romania; 4 Thoracic Surgery Department, Marius Nasta Institute of Pneumophtiziology, 050159 Bucharest, Romania

**Keywords:** hemangiopericytoma, solitary fibrous tumor, pulmonary metastases, hemoptysis, case report

## Abstract

**Background and Clinical Significance:** Solitary fibrous tumor (SFT), historically termed hemangiopericytoma (HPC), is a rare fibroblastic mesenchymal neoplasm with variable biological behavior. Pulmonary involvement is uncommon and may represent either a primary thoracic tumor or metastatic disease from an extrapulmonary site. Its clinical course ranges from indolent, surgically curable disease to aggressive malignancy with local recurrence and distant dissemination. In this retrospective case, confirmatory STAT6 immunohistochemistry was unavailable; therefore, the tumor is described as a hemangiopericytoma/solitary fibrous tumor spectrum neoplasm. **Case Presentation:** We report the case of a 33-year-old woman who presented with sudden-onset hemoptysis and was found to have two large, well-defined bilateral pulmonary masses. Initial clinical and radiological evaluation raised suspicion of primary pulmonary tumors or other benign lesions. Because both lesions were considered resectable, staged pulmonary resections were performed. Subsequent reassessment of the patient’s medical history revealed previous surgeries for a poorly documented recurrent thigh tumor, later confirmed to represent the primary malignant hemangiopericytoma/solitary fibrous tumor spectrum neoplasm. Despite staged pulmonary resections, systemic chemotherapy, and further oncologic management, the disease progressed rapidly, with cerebral, bilateral pulmonary, mediastinal, and subcutaneous metastases. The patient died within 18 months of the initial pulmonary diagnosis. **Conclusions:** This case highlights the diagnostic difficulty of metastatic pulmonary hemangiopericytoma, particularly when the primary soft tissue tumor is inadequately documented. It emphasizes the importance of detailed clinical history, retrieval of previous histopathological reports, and long-term surveillance in patients with soft tissue tumors, even when initially considered benign.

## 1. Introduction and Clinical Significance

Solitary fibrous tumor (SFT) is a rare fibroblastic mesenchymal neoplasm that may arise at virtually any anatomical site. The term “hemangiopericytoma” (HPC), initially used for tumors with a characteristic perivascular growth pattern, has historical significance; however, current classifications incorporate soft-tissue tumors previously designated as HPC within the SFT spectrum [1,2,3]. In this report, the term HPC is retained only when referring to the historical terminology used in the available pathology records. SFT exhibits heterogeneous biological behavior. Although many tumors remain localized, local recurrence and distant metastasis, including delayed relapse, may occur. Risk assessment incorporates clinicopathological variables such as patient age, tumor size, mitotic activity, and necrosis; therefore, long-term surveillance is warranted [2,3,4]. The rarity of this entity is reflected in population-based data, with a reported incidence of 0.023 per 100,000 [5]. Thoracic involvement is uncommon and may represent either a primary thoracic SFT or metastatic disease from an extrapulmonary primary. Malignant thoracic tumors may show local invasion and distant dissemination, emphasizing the importance of complete staging and prolonged follow-up [6]. Imaging is essential for assessing tumor extent and planning treatment but is not diagnostic; histopathological examination, supported by immunohistochemistry when tissue is available, remains central to diagnosis [2,3]. Complete surgical resection is the principal treatment when technically feasible. The management of locally advanced or metastatic disease should be individualized through multidisciplinary discussion because evidence supporting systemic treatment remains limited [3]. In patients presenting with unusual pulmonary masses and a history of recurrent soft-tissue tumors, active retrieval and review of prior pathology are essential to establish the origin of the pulmonary lesions. Here, we report a patient with bilateral pulmonary metastases from a previously poorly documented malignant thigh tumor, historically diagnosed as HPC, who presented with hemoptysis.

## 2. Case Presentation

A 33-year-old woman presented to the emergency department with sudden-onset hemoptysis. She had no relevant personal medical history, was a non-smoker, and reported no occupational exposure to respiratory toxins. Her family history was notable for lung cancer in her father. Emergency bronchoscopy revealed a small amount of blood within the bronchial tree bilaterally, more prominent on the left side. The left upper lobe bronchus was obstructed by a blood clot that could not be removed by bronchial toileting. Bronchoscopic biopsy was not attempted because no endobronchial lesion suitable for sampling was identified. In addition, the left upper lobe bronchus was obstructed by a blood clot, and the suspected vascular nature of the tumor raised concern regarding procedure-related bleeding. On admission, the patient was conscious and hemodynamically stable. Physical examination did not reveal signs of acute respiratory failure. Pulmonary auscultation was without focal abnormalities, and no peripheral lymphadenopathy or clinically evident soft tissue mass was identified at the time of presentation. The principal events in the patient’s clinical course, from the initial resection of the thigh tumor to the final outcome, are summarized in Table 1.

### 2.1. Diagnostic Assessment

**Figure 1:**
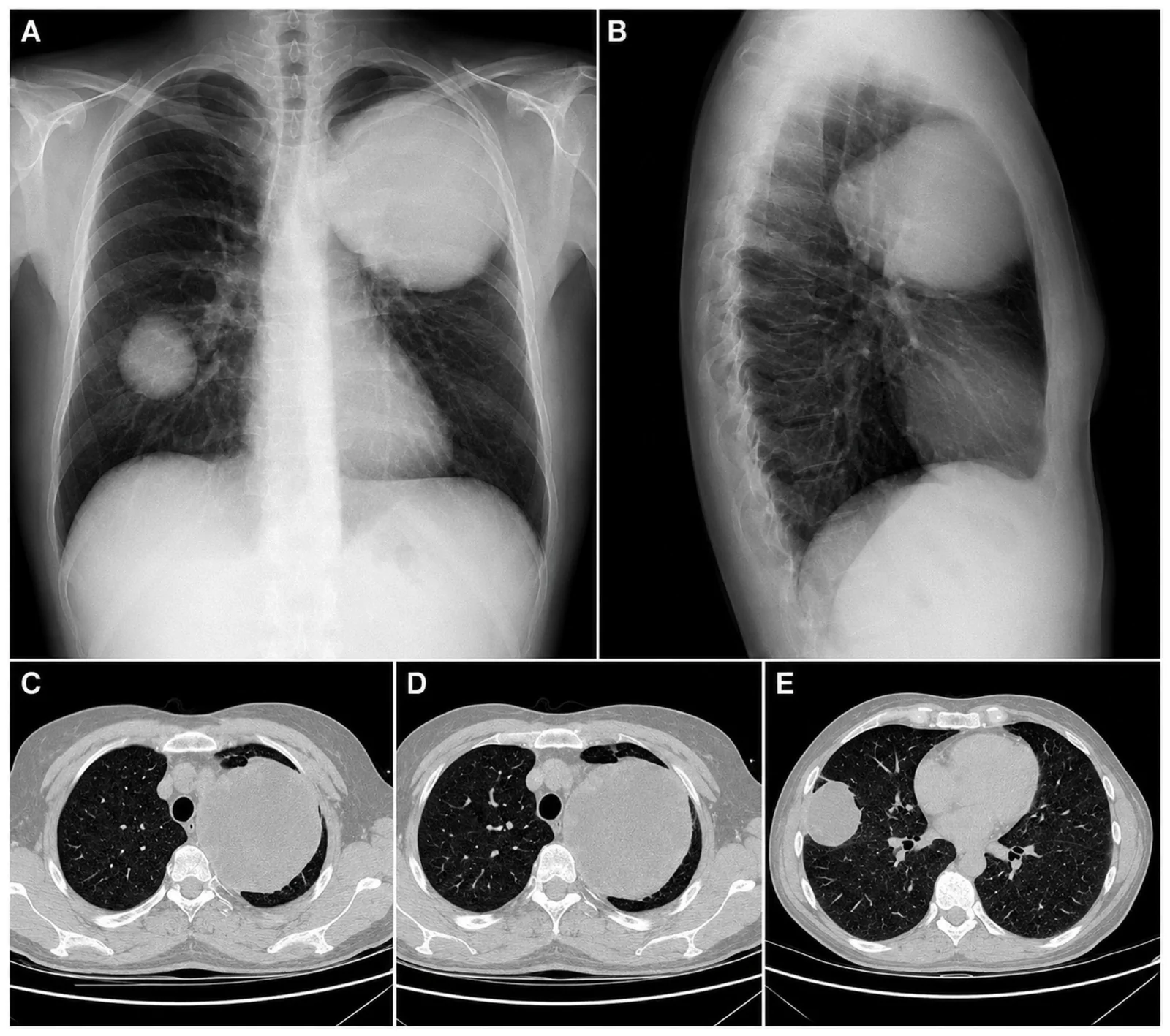
Chest radiography and computed tomography findings at initial presentation. (**A**). Posteroanterior chest radiograph showing two well-defined rounded pulmonary masses, including a large lesion projected over the left upper lung field and a smaller lesion projected over the right middle lung field. (**B**). Lateral chest radiograph confirming the posterior/apical projection of the larger thoracic mass. (**C**,**D**). Axial chest CT images showing a large solid mass in the left upper lobe/apical region, with broad pleural and mediastinal contact. (**E**). Axial chest CT image showing a second well-defined solid lesion in the right middle lobe, with pleural contact and lateral pleural involvement.

Chest radiography, including posteroanterior and lateral views, showed two well-defined rounded pulmonary masses: a large lesion of approximately 10 cm projected over the left upper lung field and a smaller lesion of approximately 4 cm projected over the right middle lung field (Figure 1A,B). Routine laboratory tests were within normal limits, and serum anti-Echinococcus antibodies were negative. The initial differential diagnosis included benign pulmonary tumors, hydatid disease, synchronous primary pulmonary tumors, and, less likely, pulmonary metastases from an undiagnosed extrapulmonary malignancy.

Chest computed tomography revealed two solid pulmonary masses: one measuring 9.1 × 9.5 × 11 cm, located in the apical segment of the left upper lobe, with invasion of the mediastinal pleura, and a second lesion measuring 5.8 × 3.5 × 5 cm, located in the right middle lobe, with lateral pleural infiltration (Figure 1C–E). No distant metastases were identified at the initial staging evaluation. Although histopathological confirmation was pending, the imaging characteristics raised strong suspicion of malignancy. The main diagnostic challenge was the absence of complete documentation regarding the patient’s previous recurrent thigh tumor at the time of pulmonary presentation. Because the prior lesion had reportedly been considered benign, metastatic hemangiopericytoma was not initially suspected. This delayed recognition of the extrapulmonary primary tumor and contributed to a broad initial differential diagnosis. At initial pulmonary staging, no distant metastases were identified; however, the presence of bilateral large pulmonary lesions and subsequent confirmation of a prior malignant thigh hemangiopericytoma supported metastatic disease with poor prognostic implications.

### 2.2. Therapeutic Intervention and Histopathological Diagnosis

**Figure 2:**
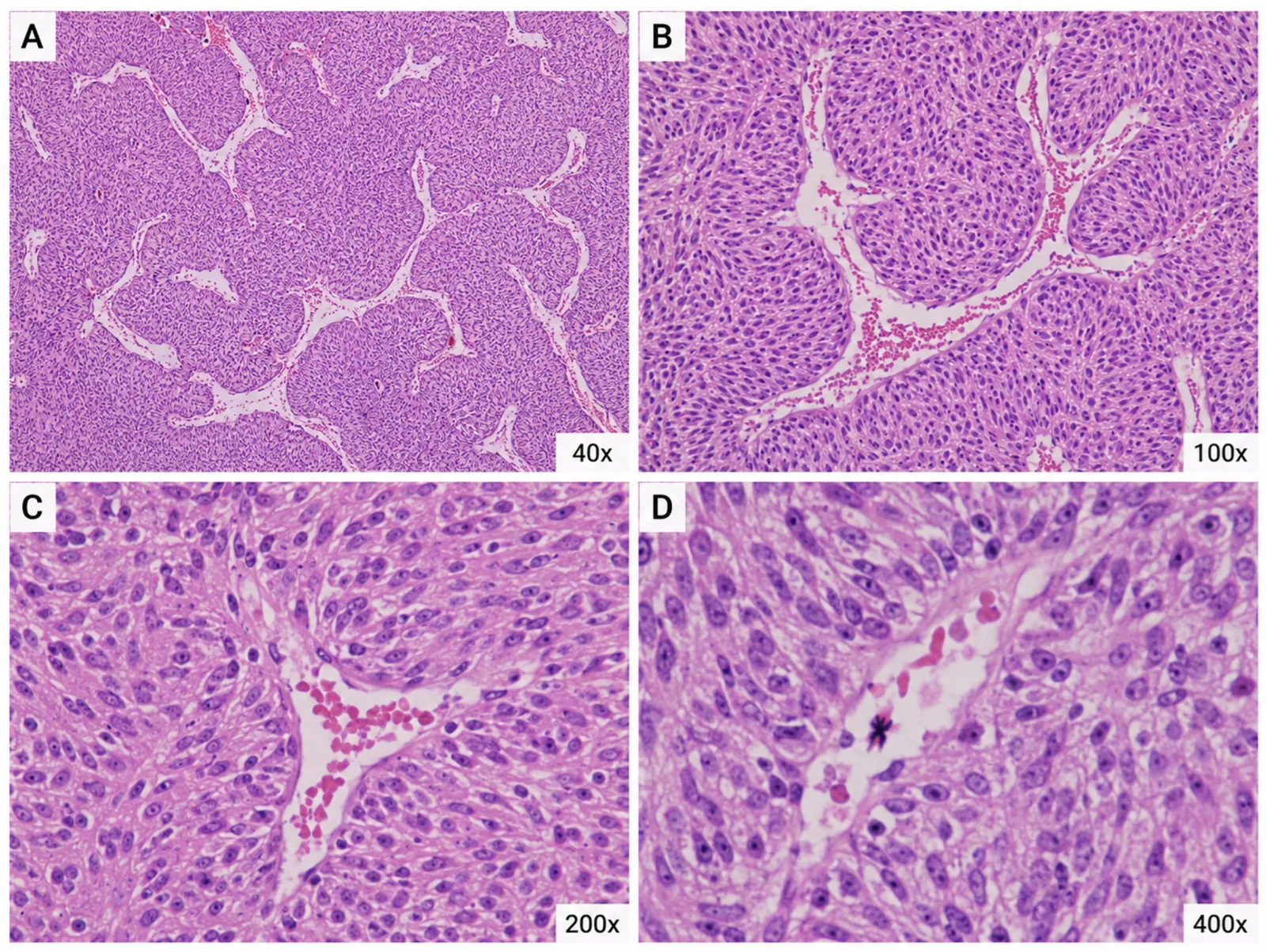
Histopathological features of malignant hemangiopericytoma/solitary fibrous tumor spectrum neoplasm. (**A**) Low-power hematoxylin and eosin staining showing a highly cellular spindle-cell tumor with prominent branching, thin-walled vascular spaces arranged in a characteristic “staghorn” pattern. (**B**) Intermediate-power view demonstrating densely packed spindle cells surrounding irregular vascular channels. (**C**) High-power view showing spindle-to-oval tumor cells with moderate nuclear atypia and increased cellularity. (**D**) High-power view (400×) showing tumor cells with moderate nuclear atypia.

Because both pulmonary lesions were considered resectable, staged pulmonary resections were performed. The first intervention consisted of left upper lobectomy. The available operative records did not specify the surgical approach, resection-margin status, lymph-node evaluation, or the extent of macroscopic pleural or mediastinal invasion. Accordingly, no inference regarding complete resection or nodal status is made. Intraoperative frozen-section examination suggested a sarcomatous tumor, and final histopathological evaluation confirmed a malignant hemangiopericytoma/solitary fibrous tumor spectrum neoplasm (Figure 2).

The histopathological findings were consistent with a malignant hemangiopericytoma/solitary fibrous tumor spectrum neoplasm. The available pathology report did not document intratumoral hemorrhage or a complete immunohistochemical profile. In particular, STAT6 immunohistochemistry was unavailable; therefore, confirmatory immunohistochemical characterization and definitive retrospective reclassification could not be performed. No specific pathological correlate for the presenting hemoptysis could be established retrospectively. Following the first pulmonary resection, the patient received six cycles of cisplatin-based systemic chemotherapy. Detailed information regarding the complete regimen, dose, treatment schedule, and rationale for treatment selection was not available in the records reviewed for this case report. An interim radiological assessment between completion of chemotherapy and right middle lobectomy was also unavailable; therefore, the radiological response of the right-sided pulmonary lesion could not be retrospectively categorized as response, stable disease, or progression. Subsequently, a right middle lobectomy was performed for the contralateral pulmonary lesion. Histopathological examination of the resected right-sided lesion also confirmed malignant hemangiopericytoma.

Because of the known recurrence and metastatic potential of this tumor type, the patient’s medical history was reassessed in detail. At that point, she disclosed two previous surgical interventions for a right thigh tumor. The first procedure, performed five years earlier, had reportedly resulted in a diagnosis of “myoma.” The second intervention, performed two years earlier for local recurrence, had no available histopathological report at the time of pulmonary diagnosis, as the patient and her family had not pursued further documentation, assuming the lesion was benign. After the second pulmonary surgery, the previous pathology report was retrieved and confirmed that the thigh lesion had been a malignant hemangiopericytoma. The bilateral pulmonary tumors were therefore interpreted as metastatic lesions originating from the previously treated soft tissue tumor.

### 2.3. Follow-Up and Outcomes

Despite aggressive surgical and oncological management, the disease progressed rapidly. Within one year, the patient developed frontal and occipital cerebral metastases, multiple bilateral pulmonary and mediastinal lesions, and a subcutaneous metastasis. Further oncological management was attempted; however, the disease followed an aggressive course with rapid systemic dissemination. The patient died within 18 months of the initial pulmonary diagnosis.

## 3. Discussion

Solitary fibrous tumor (SFT), historically termed hemangiopericytoma (HPC), is a rare fibroblastic mesenchymal neoplasm with heterogeneous biological behavior. Current WHO classification incorporates soft-tissue tumors historically designated as HPC within the SFT spectrum. The risk of local or distant recurrence is variable and may persist over a prolonged period; risk assessment is based on clinicopathological factors including patient age, tumor size, mitotic activity, and necrosis [2,3,4].

Pulmonary involvement may represent a primary thoracic tumor or metastatic disease from an extrapulmonary SFT. In the present patient, retrieval of the previous thigh pathology report was decisive in establishing the metastatic origin of the bilateral pulmonary lesions. This diagnostic step is particularly important when imaging is non-specific and the initial history of a recurrent soft-tissue tumor is incomplete. In a study of 213 patients with cerebral hemangiopericytoma followed for up to 372 months, the most frequent metastatic sites were bone, reported in 19.6% of cases; pleura and lung, in 18.4%; liver, in 17.6%; and vertebrae, in 14.1% [7]. Primary pulmonary hemangiopericytoma is even rarer, with only a limited number of cases described in the literature. These tumors are often minimally symptomatic; however, when present, hemoptysis and pain have been considered unfavorable prognostic indicators. Radiologically, endobronchial growth, reported in approximately 10% of cases, may be associated with a more favorable prognosis [8,9].

In Romania, only one case of primary pulmonary hemangiopericytoma with fatal outcome has previously been reported in the medical literature [6]. To our knowledge, the present case represents the first documented Romanian case of metastatic pulmonary hemangiopericytoma. The diagnostic difficulty was mainly related to the absence of initial clinical suspicion, as the patient did not initially report the full history of her previous recurrent thigh tumor, and the prior histopathological documentation was not available at the time of pulmonary presentation.

The initial presentation with hemoptysis in a young patient from a tuberculosis-endemic country first raised the possibility of pulmonary tuberculosis. However, chest radiography revealed two well-defined pulmonary masses, shifting the differential diagnosis toward hydatid disease, benign pulmonary tumors, or synchronous primary lung tumors. The CT findings, particularly the presence of large bilateral solid lesions with pleural involvement, further supported the suspicion of malignancy. Given the patient’s young age, non-smoking status, and absence of occupational exposure, synchronous primary lung adenocarcinomas were also considered in the differential diagnosis.

Definitive diagnosis was established only after histopathological examination of the resected pulmonary lesions. This finding prompted a more detailed reassessment of the patient’s medical history and led to retrieval of the previous thigh tumor pathology report. The final interpretation was metastatic malignant hemangiopericytoma originating from a previously undertreated and insufficiently documented soft tissue tumor.

The role of systemic therapy in advanced hemangiopericytoma/solitary fibrous tumor spectrum neoplasms remains uncertain and should be individualized. In the present case, six cycles of cisplatin-based systemic chemotherapy were administered; however, detailed regimen and rationale data were unavailable, and the subsequent clinical course remained aggressive despite multimodal treatment. The absence of interim radiological assessment precluded retrospective categorization of treatment response. The principal contribution of this case is the diagnostic delay caused by incomplete documentation of a recurrent soft tissue tumor. In patients presenting with unexplained bilateral pulmonary masses and a history of recurrent soft tissue lesions, even when previously considered benign, earlier histopathological reports should be actively retrieved and reviewed. This case also emphasizes that imaging alone may not establish the diagnosis, that long-term surveillance is essential after recurrent soft tissue tumors, and that management of rare aggressive tumors requires individualized multidisciplinary decision-making.

The strength of this case report lies in the histopathological confirmation of both pulmonary lesions and the subsequent retrieval of the previous thigh tumor pathology report, which clarified the metastatic origin of the disease. The case also illustrates a clinically relevant diagnostic pitfall in patients with recurrent soft tissue tumors and unusual pulmonary masses. The main limitations are its retrospective nature, incomplete initial documentation of the primary thigh tumor, limited availability of detailed bronchoscopy and operative records, lack of an interim radiological assessment after chemotherapy, unavailability of a complete immunohistochemical profile including STAT6, incomplete details regarding chemotherapy regimen, dosing, treatment rationale, and tolerability, and the inability to obtain the patient’s perspective because of the fatal outcome.

## 4. Conclusions

This case highlights the diagnostic and therapeutic complexity of malignant hemangiopericytoma with pulmonary metastases, a clinical entity so rare that it may not be considered during the initial diagnostic work-up. In the present case, delayed recognition of a previous recurrent soft tissue tumor, together with the non-specific presentation of hemoptysis in a tuberculosis-endemic region, contributed to a challenging diagnostic pathway.

Despite staged pulmonary resections and systemic chemotherapy, the disease followed an aggressive course characterized by early recurrence and widespread dissemination, including cerebral, bilateral pulmonary, mediastinal, and subcutaneous metastases. This evolution underlines the unpredictable biological behavior of malignant hemangiopericytoma and the limited efficacy of currently available systemic treatment options in advanced disease.

Our experience emphasizes the importance of detailed medical history, systematic retrieval of previous histopathological reports, especially in patients with recurrent soft tissue tumors, and long-term surveillance for metastatic disease. International collaboration, dedicated case registries, and prospective studies are needed to better define evidence-based diagnostic and therapeutic algorithms for this rare and challenging tumor.

## Figures and Tables

**Table 1:** Timeline of the clinical course.

Time Point	Clinical Event
Five years before pulmonary presentation	First resection of a right thigh tumor, reportedly diagnosed as “myoma”.
Two years before pulmonary presentation	Repeat resection for local recurrence of the right thigh tumor; the pathology report was unavailable at pulmonary presentation.
Initial pulmonary presentation	Sudden-onset hemoptysis prompted emergency evaluation and bronchoscopy.
Initial staging	Chest radiography and CT showed two large bilateral pulmonary masses.
First thoracic intervention	Left upper lobectomy; histopathology confirmed the malignant tumor.
Postoperative treatment	Six cycles of cisplatin-based systemic chemotherapy were administered.
Second thoracic intervention	Right middle lobectomy; histopathology confirmed the same diagnosis.
Subsequent reassessment	The previous thigh pathology report was retrieved, confirming the primary tumor and metastatic pulmonary disease.
Within one year	Cerebral, bilateral pulmonary, mediastinal, and subcutaneous metastases developed.
Eighteen months after initial pulmonary diagnosis	Death from progressive metastatic disease.

## Data Availability

The data presented in this study are available on request from the corresponding author due to patient confidentiality.

## References

[1] Stout A.P. (1949). Hemangiopericytoma—A Study of Twenty-five New Cases. Cancer.

[2] Sbaraglia M., Bellan E., Dei Tos A.P. (2021). The 2020 WHO Classification of Soft Tissue Tumours: News and perspectives. Pathologica.

[3] Martin-Broto J., Mondaza-Hernandez J.L., Moura D.S., Hindi N. (2021). A Comprehensive Review on Solitary Fibrous Tumor: New Insights for New Horizons. Cancers.

[4] Salas S., Resseguier N., Blay J.Y., Le Cesne A., Italiano A., Chevreau C., Rosset P., Isambert N., Soulie P., Cupissol D. (2017). Prediction of local and metastatic recurrence in solitary fibrous tumor: Construction of a risk calculator in a multicenter cohort from the French Sarcoma Group (FSG) database. Ann. Oncol..

[5] Wang K., Mei F., Wu S., Tan Z. (2020). Research Article Hemangiopericytoma: Incidence, Treatment, and Prognosis Analysis Based on SEER Database. BioMed Res. Int..

[6] Radulescu D., Pripon S., Ciuleanu T.E., Radulescu L.I. (2007). Malignant Primary Pulmonary Tumor with Hemangiopericytoma-Like Features: Conventional Hemangiopericytoma Versus Solitary Fibrous Tumor. Clin. Lung Cancer.

[7] Ratneswaren T., Hogg F.R.A., Gallagher M.J., Ashkan K. (2018). Surveillance for metastatic hemangiopericytoma-solitary fibrous tumors-systematic literature review on incidence, predictors and diagnosis of extra-cranial disease. J. Neuro-Oncol..

[8] Halle M., Blum U., Dinkel E., Brugger W. (1993). CT and MR Features of Primary Pulmonary Hemangiopericytomas. J. Comput. Assist. Tomogr..

[9] Rusch V.W., Shuman W.P., Schmidt R., Laramore G.E. (1989). Massive Pulmonary Hemangiopericytoma An Innovative Approach to Evaluation and Treatment. Cancer.

